# Midwives’ knowledge and attitudes when encountering Gender-Based Violence in their practice at a maternity-hospital in Kingston, Jamaica

**DOI:** 10.3402/qhw.v11.29358

**Published:** 2016-02-15

**Authors:** Cynthia Pearl Pitter

**Affiliations:** The UWI School of Nursing Mona, Faculty of Medical Sciences, University of the West Indies, Kingston, Jamaica

**Keywords:** Gender-based violence, knowledge, attitudes and practices, midwives

## Abstract

**Introduction:**

Gender-based violence (GBV) can have devastating consequences for pregnancy because both mother and child are at risk. Midwives are in a strategic position to identify and empower pregnant women experiencing GBV; however, currently midwives in Jamaica are not required to screen for GBV, neither are they prepared to do so.

**Aim:**

This study forms the baseline of a larger study designed to improve the capacity of midwives to identify and treat pregnant women experiencing GBV in Jamaica. This specific component assessed midwives’ knowledge and attitudes when encountering GBV in their practice in Kingston, Jamaica.

**Methods:**

A qualitative study design was used. Six practicing midwives were purposely selected to participate in a focus group discussion at the antenatal clinic of a hospital in Kingston, Jamaica.

**Results:**

All six respondents said it was very important to screen for GBV among pregnant women in their care. The findings from their report revolved around six themes, namely midwives have suboptimal knowledge, are exposed to women experiencing GBV in pregnancy, lack professional preparedness, report gaps in the institutional framework to guide their practice, are concerned for their safety and security, and are willing to intervene.

**Conclusion:**

This study confirmed that midwives are aware of the problem and are willing to intervene but are faced with lack of formal procedures to detect and treat pregnant women who are experiencing GBV. Findings could be used to inform a protocol which is being developed to guide midwives’ practice. Findings could also be incorporated in the national strategy to eliminate GBV.

Midwives as reproductive health practitioners are in a strategic position to mitigate intergenerational violence by identifying and making the appropriate referrals for pregnant women who are victims of gender-based violence (GBV) (Taket et al., 2003). Studies have shown that up to 32.5% of all pregnant women, especially those in developing countries, experience GBV (Ezeanoche, Olagbuji, Ande, Kubeyinje, & Okonofua, 2011; Nasir & Hyder, 2003). In Jamaica, where this study was conducted, Pitter and Dunn (2015, unpublished study) found that 36% of pregnant women are being physically, emotionally, financially, and/or sexually abused by their intimate partners. The majority of women who experience GBV will not disclose their abuse unless they are asked directly (Burris & Jaffe, 1984). However, some women in this situation wish that [health care providers] would ask them about it (Scheffer Lindgre & Renck, 2008).

GBV in the general population can lead to homicides, suicides, or result in negative health outcomes such as HIV infection, as well as unwanted and unplanned pregnancies for women (Dunkle et al., 2004). GBV during pregnancy increases the risk of the pregnancy becoming “high risk”, leading to miscarriages, stillbirths, or premature labour (Lazenbatt & Thompson-Cree, 2009; The Royal College of Midwives, 1999).

Midwives are entrusted to monitor pregnant women to ensure safe pregnancies and deliveries. They are in a strategic position to successfully manage GBV and are among the first to see victims and survivors of such violence [Fraser & Cooper, 2009; Lauti & Miller, 2008; The Forty-Ninth World Health Assembly (WHA 49.25), 1996]. Mezey, Bacchus, Haworth, and Bewley (2003) agreed that midwives are aware of their responsibilities to women experiencing GBV but they rarely enquire about abuse. Researchers like Lazenbatt and Thompson-Cree (2009); the Royal College of Midwives (1999); Baachus, Mezey, Bewley, and Haworth (2004) agreed with Mezey et al. (2003) that there are many contributing factors that are preventing midwives from enquiring about GBV. Among the factors reported by midwives were lack of time, lack of confidence, having safety concerns, shortage of staff, low staff morale, personal experiences with GBV, and lack of professional preparedness and formal procedures to guide their practice.

The current study which is being done in Jamaica has revealed that Jamaican midwives are not required by law to screen for or report cases of GBV. It has also emerged that many cases of pregnant women experiencing GBV are not detected. The lack of screening for GBV by midwives may be a factor contributing to Jamaica's inability to achieve Millennium Development Goals (MDGs) #3–5, which promote gender equality, the empowerment of women, and child and maternal health.

Little is known about Jamaican midwives’ knowledge and attitudes when encountering GBV in their practice and how these may be influencing pregnant women's willingness to disclose their experiences in antenatal clinics. The study has enabled midwives to identify the gaps while empowering them to become part of the process to eliminate GBV in Jamaica.

## Theoretical perspectives

The absence of systems for GBV screening in the health sector can be explained by patriarchy as a reflection of a system of unequal power. Feminists argue that patriarchy is an institution or system that promotes dominance and control by men (Wilson, 2000). Fraser and Cooper (2009) argued that issues that mainly affect women, such as screening for GBV are not considered to be a priority within large institutions where the management team is mainly male dominated. The absence of screening for GBV in maternity care could be regarded not only as a reflection of patriarchy in the health system but also a form of “institutional silence” on GBV. Professions dominated by females such as midwifery are historically considered subordinate to medical doctors the, majority of whom were men, in a field that was traditionally male dominated. Midwives are among the 80% of women employed in the health care system but as a group seem to have less power at the highest levels of decision-making in the health system (World Health Organization, 2009). This is significant because male dominance may eliminate the voice of those employed at the lower levels, the majority of whom are females. The medical model that is practiced in Jamaica's health care system may be viewed as one that obscures rather than elucidates knowledge of abuse and one that puts [midwives] under institutional constraints (Warshaw, 1989).

The current study has recognized that midwives are ideally placed, as agents of change that can emancipate and empower not only women who are experiencing abuse but also professional midwives who may also be experiencing abuse. The time has come for the campaign against GBV to be broadened to include the health care system, to value the role of midwives, and to encourage them to be more responsive to issues, such as GBV, that affect women. Central to this discourse however is how midwives as women view themselves as part of the solution rather than victims of circumstance. Their views can influence their ability to empower their clients who are experiencing GBV. The findings from this study could provide valuable insight to guide midwives’ practice in the therapeutic management of GBV among pregnant women.

### Study aim

This study forms the baseline of a larger study designed to improve the capacity of midwives to identify and treat pregnant women experiencing GBV in Jamaica. This specific component assessed midwives’ knowledge and attitudes when encountering GBV in their practice at a maternity hospital in Kingston, Jamaica.

## Methodology

### Research design

This qualitative study design was used as part of a participatory action research study to explore midwives’ knowledge and attitudes when encountering GBV in their practice at the antenatal clinic of a hospital in Kingston, Jamaica. The aim was to establish collaborative and non-exploitative relationships and to conduct research that is transformative (Creswell, 2008). Central to this research process is the framework which is embedded in the absence and invisibility of midwives as women to see themselves as “knowers”. Mezey et al. (2003) have also used the qualitative approach to ascertain the midwives’ perceptions and experiences of routine enquiry for domestic violence (DV). A focus group discussion was also used for the collection of rich narratives from this small number of midwives working in the antenatal clinic (Creswell, 2008).

### Study setting

The study was conducted at a public hospital that offers maternity care in Kingston, Jamaica. The hospital operates as a teaching facility for midwives and serves clients from different social and economic backgrounds. It is one of two maternity hospitals that accept referrals for high-risk patients from the 14 parishes in Jamaica. Each year, this hospital accounts for almost 3000 of the 40,000 births island wide. The antenatal clinic serves more than 600 pregnant women per month. These pregnant women are booked at 8–10 weeks’ gestation and are cared for up to 6 weeks post-delivery. The clinic has a staff compliment of six midwives.

### Sampling technique

Purposive sampling technique was used to select all six midwives that met the selection criteria. Creswell (2008), Polit and Beck (2008), and Robson (2011) recommended that 6–12 persons are ideal for a focus group discussion. However, Côté-Arsenault and Morrison-Beedy (1999) have suggested that 4–5 persons are acceptable when discussing emotional or sensitive topics such as GBV. The midwives were intentionally selected because they were rich with information based on their knowledge, working experience, and position in the clinic (Creswell, 2008; Robson, 2011), and often they were the first points of contact for the pregnant women seeking care at the institution. Midwives who met the inclusion criteria were those who had at least 6 months uninterrupted working experience in the antenatal clinic. Pupil midwives were not included in the sample and midwives on temporary assigned were excluded.

### Data collection procedure and instrument

Data were collected once in November 2014 through a focus group discussion using a structured interview guide with six midwives assigned to the antenatal clinic in a maternity hospital in Kingston, Jamaica. Contact was made with the midwives after obtaining approval from the institution's authority. Participants were informed of the purpose of the study 4 weeks in advance and a date was agreed upon for the discussion to be held. This was followed up with a written invitation and telephone calls as reminders. On the day of the focus group discussion, midwives independently provided written informed consent for their participation. Confidentiality procedures were outlined and midwives were made aware that their participation was voluntary. They were also advised that they had the option to refuse or withdraw from the discussion at any time. Midwives also completed brief demographic questionnaires. The focus group included a facilitator and note taker. The semi-structured interview guide included open-ended questions which explored four areas: knowledge of GBV, midwives perceived role of GBV, midwives practice at the institution, and their willingness to participate in the development and implementation of a protocol for screening for GBV among their clients. The interview lasted 60 min and was audiotaped.

### Data analysis

Audiotaped data were transcribed verbatim and were analysed using content analysis. Data were read to obtain a preliminary understanding of the text. The text was reread several times while playing the taped interviews, to ensure accuracy of the data transcribed. Field notes and observations of non-verbal behaviours were incorporated into the data. Various themes emerged and were coded into major themes and subthemes, then interpreted and summarized. A systematic sorting of the data (Polit & Hungler, 1995) helps to theorize that the medical model that is practiced in Jamaica's health care system is one that could obscure rather than elucidate knowledge of abuse and one that puts [midwives] under institutional constraints (Warshaw, 1989).

### Ethical consideration

Ethical approval was received from the local institutional review boards. Permission was sought and granted from the chief executive officer, directors of nursing, and the charge nurse at the hospital. Each participant gave consent by completing the informed consent form and had the opportunity to refuse after a brief overview of the study. The participants were asked to complete demographic data sheets before the start of the discussion. The required steps were observed to ensure the rights of participants were acknowledged and safeguarded throughout the study. The midwives were assured that their responses would be kept confidential. The recorded discussion was stored on a personal computer with a password and kept for audit after data analysis. To ensure confidentiality, each participant was assigned a name and referred by that name during the discussion. In addition, the Reproductive Health Survey (RHS) (2008) found that 35.4% of ever-partnered women aged 15–45 years in Jamaica have been abused by their intimate partner. Therefore, it is highly likely that some midwives who have participated in the study may have had or are currently experiencing GBV. In keeping with Lazenbatt and Thompson-Cree's (2009) recommendation, each midwife was given the telephone number of a 24-h DV hotline at the end of the discussion.

## Research findings

### Demographic profile of midwives

The age of the six midwives who participated in the focus group discussion ranged from 28 to 46 years and over. They share among themselves 36 years of experience as midwives and they have 6 months to 11 years working experience in the clinic. Three midwives were trained at the baccalaureate level and the other three at the certificate level.

### Emerging themes

Six major themes emerged from the data, namely midwives have suboptimal knowledge, midwives are exposed to women experiencing GBV in pregnancy, midwives lack professional preparedness, there are gaps in the institutional framework to guide practice, midwives are concerned for their safety and security, and midwives are willing to intervene.

### Theme 1: Midwives have suboptimal knowledge of GBV

All six midwives reported that they have never received formal training but are aware of some of the issues surrounding GBV. Some midwives reported that GBV is very prevalent and affects everyone, but women are more susceptible. They also stated that GBV has more health consequences for pregnant women. According to the midwives some women are of the view that abuse from an intimate partner means “love”. The midwives reported that some women will remain in the situation because of lack of social support whereas others will end the relationship after the birth of the baby or when the children are all grown up. Some midwives are of the view that secrecy is a hindrance and is preventing meaningful interventions for women who are being abused as shown by the quotes below:

Gender-Based Violence is treated like private business … (Anna)

Some persons are afraid they might get hurt … women get hurt even if reported to the police … not enough attention is given to it. (Bell)

It is a big problem in our society … often swept under the carpet. (Tiana)

However, despite their awareness of GBV, they have also identified and reported that they do not know how to effectively respond to women who are in crisis.

### Theme 2: Midwives are being exposed to women experiencing GBV in pregnancy

Despite midwives not being trained, they are still facing the challenge of having to attend to pregnant women who are experiencing GBV. Three midwives recalled their encounters with pregnant women who were experiencing GBV on several occasions. Here are some of their experiences that they shared:

The woman became suicidal because her partner didn't want her to get pregnant … she was kicked out of the house. I'm not sure how it ended. (Cindy)

In my case the lady's husband died and left her with a lot of money … she fell in love with a younger man who wanted her to sign over all the business to him. He cheated on her. She had to see psychiatrist and social worker. The case resulted in her getting over her lover and vowing that she would live by herself with her baby. (Anna)

It was mostly emotional with one or two postnatal women. Half of the cases are unwanted pregnancies and their spouses had suggested abortion. There was constant degrading of the person, ignoring the child, lack of financial support resulting in psychological trauma, to the point where some had suicidal thoughts and depression after the baby was born. I remembered a lady had a miscarriage shortly after her beating but I am not sure if the miscarriage was from the beating. (Tiana)

### Theme 3: Lack of professional preparedness

The lack of professional preparedness created lack of confidence and uncertainty among midwives regarding their practice. When the midwives were asked if they were trained to manage GBV, most acknowledged that they had not received formal training in the management of GBV by the institution or during their span of studies. The midwives reported that their knowledge of GBV came from society, the media, or their own readings. All midwives were concerned about the negative health effects of GBV during pregnancy. They agreed that training would give them confidence and prepare them to screen and intervene on behalf of pregnant women who are at risk or experiencing GBV.

Training will give us confidence. If and when we are faced with certain situations we would know exactly what to do. (Elsa)

Training is a good idea … I am ready to be a part of the first batch … information is power. However, training must correlate with other relevant agencies such as the medical social worker. (Tiana)

We should be certified in this area. We would be better prepared to get involved and make a difference. (Cindy)

### Theme 4: Gaps in institutional framework to guide practice

The study identified that there is some level of inconsistency in the reporting of GBV among midwives in the antenatal clinic. Only two midwives attempted to answer when they were asked about the mechanisms for reporting GBV at their health institution. These two midwives reported that the patients are referred to the police or to the social worker depending on the case. One midwife reported that it is mandatory that teenagers below the age of consent are referred to the social worker or police; however, the midwives were uncertain as to who should report these cases to the police. In addition, the midwives were concerned that there is no opportunity for midwives to follow up with these women. Midwives believe that they have a moral obligation to report the crime of GBV even though the institution does not require her to report.

In addition to the inconsistency for the reporting of GBV, the midwives reported that there is a lack of resources. A midwife who has been working in the clinic shared some of the challenges she faced as a result of a lack of resources to support pregnant women who are experiencing GBV as follows:

As midwives we are exposed to pregnant women who are been abused. There are times when we want to help those persons but there are no resources such as finances and not enough medical social workers that we can refer these women to. (Cindy)

### Theme 5: Safety and security concerns

Safety and security emerged as a theme although the midwives were not asked directly about it. Instead they were asked what they think should be included in the content of a proposed manual to guide practice. All six midwives proposed that safety and security should be included in the proposal. Midwives are very concerned for their safety and security as well as those they care for. Here are some of their views:

There is a great need for the protection for the family or for the woman especially when children are involved. We need to protect them. (Cindy)

The proposal should cover protection for the midwives and the health team. (Tiana)

One midwife believes that the protection of their safety and security could empower them and give them the right to intervene.

### Theme 6: Midwives are willing to intervene

#### Midwives’ perceived role in responding to pregnant women who are experiencing GBV

All midwives reported that they do have a role in identifying GBV among pregnant women and consider themselves to be advocates. They believe that they are morally obligated to report GBV although it is not mandated by law or required by the hospital. They strongly believe that they should have an input in the development of a proposed protocol to guide their practice. In addition, they believe that brochures with GBV information should be available to pregnant women. The midwives also believe that separate and apart from asking the patients, there are other covert signs to identify those at risk, such as improperly dressed, unkempt, depressed, elevated vital signs, not sleeping, and obvious physical trauma. The midwives reported that there are other indirect signs such as:

A controlling partner wants to know everything and follows his spouse around. He may be controlling and she doesn't want to say it in front of him. You might pick up on that one. (Belle)

Absenteeism from clinic, or whenever the woman comes she might be a bit withdrawn. The woman may display abusive behaviour toward the staff or even to her children that accompany her to the clinic. (Elsa)

Some of them tell-tales such as hitting up in the door … these things should be investigated. (Tiana)

The midwives have also reported that they are willing to be trained and participate in the development of a protocol to screen for GBV in maternity care.

## Discussion

This is a groundbreaking study conducted in Jamaica which has highlighted the knowledge and attitude of midwives when encountering GBV in their practice. With a prevalence of 34.5% of ever-partnered women experiencing GBV in the general population (RHS, 2008) and a prevalence of 36% among pregnant women (Pitter & Dunn, 2015, unpublished data), GBV in Jamaica by all accounts is a serious and widespread crime. Still many cases continue to go unreported and undetected mainly because GBV is intrinsically linked to the social and cultural norms, stigma, and fear of retribution or for further violence (Country Information and Guidance, 2015). Some of these reasons occur in the context of gender inequality and specific cultural beliefs and attitudes about gender roles, especially those concerning male and female sexuality and a pattern of economic inequality United States Agency for International Development (USAID, n.d.-a). The health care systems in many jurisdictions have proven to be successful in identifying and treating victims of GBV (USAID, n.d.-b). However, there seems to be an oversight in Jamaica's health care system, especially in maternity care. This could be viewed as an indictment for Jamaica to achieve several international goals which seek to eliminate violence against women (VAW), for which Jamaica is signatory to.

All midwives in this study have reported that they have some insight on issues surrounding GBV but in the final analysis they have admitted that they do not have the competence or confidence to effectively respond to pregnant women in crisis. Neither are these midwives professionally prepared nor have they any institutional framework to guide their practice. The midwives believe that training in GBV will increase the rate of detection in maternity settings, thereby providing an opportunity for women to access help early (Bacchus et al., 2004). Although there may be widespread concerns for mandatory screening for GBV in health care (Jezierski, 1999), the Maternal and Child Health Care for Vulnerable Mothers project has found that having a clinical pathway and guidelines will provide greater opportunity for GBV discussions to occur (Taft et al., 2015). Moreover, there is a growing consensus that providing opportunities in maternity centres to enquiry about GBV will heighten the awareness of GBV among women (Undie, Maternowska, Mak'anyengo, & Askew, 2015).

The World Health Organization (2006) has called for the integration of gender as it relates to violence into the curricula for health professionals as it is widely believed that these professionals are uniquely placed to address issues such as GBV. The problem is that GBV is not included in the educational curricula of health professionals in some schools (Keeling, 2002) such as midwifery. Therefore, it is not surprising that midwives in Jamaica would lack the knowledge and confidence to initiate or engage discussions on this important topic among their clientele.

Midwives are in a position of great respect and influence (RHS, 2008); they are on the front line for reducing intergenerational violence by identifying and treating pregnant women who are experiencing GBV (WHA 49.25, 1996). This study has found that midwives are exposed to women in crisis despite not been equipped to address the issue of GBV. This has left the midwives to draw conclusions about who they perceived to be experiencing abuse by observing the behaviours of clients and/or the interactions of clients with their spouses. When faced with such challenges, the midwives are unsure as to what they are to report and to whom they are to report cases of GBV to; on many occasions, midwives wondered what had happened to their clients and were left with many unanswered concerns (McCosker-Howard, Kain, Anderson, & Webster, 2005). This practice is contrary to the American Association of Colleges of Nursing (AACN) (1999) best practice guidelines for nurses in responding to GBV. These guidelines include the acknowledgment of the problem, the utilization of assessment skills to identify and document abuse and its health effects, legal and ethical issues in treating and reporting, and activities to prevent domestic violence. The AACN guidelines compliment the recommendation from USAID (n.d.-b) that urged the health systems to respond with linkages to legal and social services to support women survivors of violence.

Despite all the challenges, midwives in this study and other studies remain committed; they feel morally obligated and are willing to intervene on behalf of their clients (Lazenbatt & Thompson-Cree, 2009; McCosker-Howard et al., 2005). This is in contrast to the Mezey et al. (2003) groundbreaking study which found that despite being trained, midwives were not keen on screening for GBV. Some of the reasons they gave were lack of motivation and enthusiasm, insufficient time, and screening for GBV was added workload. This was not the case in this study as the midwives interviewed had a positive attitude; they remain hopeful and viewed GBV as a priority that needs urgent attention. I am suggesting that strategies to reinforce such positive attitudes should include measures to manage difficult conversations as well as ensure professional development of midwives for them to be better at responding to women in crisis. Mezey et al. (2003) warned that although midwives have caring attitudes and feel morally obligated to intervene on behalf of their clients, the perceived barriers in a busy maternity service should not be overlooked as these could limit the encounter when the woman is distressed.

Although the midwives were not asked directly about safety and security issues, all are cognizant of the threats and the impact this could have on the lives of midwives and those they care for. This finding underscores the Mezey et al. (2003) warning that these issues should not be undermined as this could lead to some potentially dangerous and certainly threatening encounters with irate partners, especially when the women attempt to seek help. These factors should not be overlooked by our local authority given the high incidence of violence in our country. Fortunately, these challenges however have not prevented midwives in this study from intervening on behalf of clients that are reporting GBV.

The lack of screening for GBV in the antenatal clinic could be seen as a lost opportunity for midwives to break the cycle of violence. I believe that midwives should capitalize on this window of opportunity to intervene and advocate for their clients and not to delay the process because of a lack of framework to guide practice. This can be accomplished by raising the awareness of GBV among the users of the clinic, targeting both mothers and their partners, and utilizing the referral system to identify pregnant women who are experiencing or at risk for GBV.

The overwhelming evidence has demonstrated that training of midwives to address culturally sensitive issues such as GBV should be top priority as it will help protect the lives of the pregnant women and their infants. This proposed approach calls for midwives who are predominately women providing the evidence to be included at the decision making table and get their voices heard.

## Conclusion

This is the first study in Jamaica that highlights midwives’ thoughts and voices as well as sheds light on such a neglected but important issue that they encounter while giving care. A great deal of focus has been placed on achieving the MDGs #4–5; however, what is repeatedly overlooked is a methodology to identify GBV in maternity centres in Jamaica. Given the physical and mental health manifestations of intimate partner abuse, it is very important that the health system remains an important component of a coordinated response to GBV (Laing, 2003). The findings of the study are an important link in the prevention framework to eliminate VAW and the call for action. The study also emphasized that midwives are central in any multisectoral response to eliminate GBV among pregnant women. Asking about GBV in pregnancy is an important step for midwives to address this national priority. The study further confirmed midwives’ awareness of the issue, but their capacity to intervene is hindered by their lack of professional preparedness and institutional framework to guide their practice. Considering these factors could support a climate of change and provide support for both midwives and pregnant women. Training midwives in GBV should be a priority not just for this institution but for maternity care services in Jamaica. This could lead to raising the awareness and effects of GBV in pregnancy and assist pregnant women to examine their relationship and their help-seeking behaviour.

### Relevance

Findings from this study will be incorporated with the findings from the larger study to inform midwifery education and practice in Jamaica. The findings will also be used to inform a screening protocol in maternity care. This could also broaden the discussion on the effects of GBV in pregnancy on the MDGs as it relates to the inability of Jamaica to significantly reduce the maternal and infant mortality rates. Findings could be incorporated in the national strategies to support linkages between law enforcement, health services, and other services to eliminate GBV in Jamaica.

### Limitations

Although findings from this study provide important information on the knowledge and attitude of midwives when encountering GBV in their practice, limitations to the study do exist. The sample included only a limited number of midwives in an antenatal clinic in one Kingston-based hospital. This limits the number of midwives in the institution and more so other maternity centres in Jamaica. Another major limitation of the study was that no information from the pregnant women was included in the study. The results of this study should be interpreted carefully because they are derived from this single preliminary investigation.
